# The Nature Index: A General Framework for Synthesizing Knowledge on the State of Biodiversity

**DOI:** 10.1371/journal.pone.0018930

**Published:** 2011-04-22

**Authors:** Grégoire Certain, Olav Skarpaas, Jarle-Werner Bjerke, Erik Framstad, Markus Lindholm, Jan-Erik Nilsen, Ann Norderhaug, Eivind Oug, Hans-Christian Pedersen, Ann-Kristin Schartau, Gro I. van der Meeren, Iulie Aslaksen, Steinar Engen, Per-Arild Garnåsjordet, Pål Kvaløy, Magnar Lillegård, Nigel G. Yoccoz, Signe Nybø

**Affiliations:** 1 Norwegian Institute for Nature Research (NINA), Trondheim, Norway; 2 Norwegian Institute for Nature Research (NINA), Oslo, Norway; 3 Norwegian Institute for Nature Research (NINA), Tromsø, Norway; 4 Norwegian Institute for Water Research (NIVA), Oslo, Norway; 5 The Norwegian Forest and Landscape Institute, Ås, Norway; 6 Norwegian Institute for Agricultural and Environmental Research (BIOFORSK), Stjørdal, Norway; 7 Institute for Marine Research (IMR), Bergen, Norway; 8 Statistics Norway (SSB), Oslo, Norway; 9 Department of Mathematical Sciences, Centre for Conservation Biology, Trondheim, Norway; 10 University of Tromsø (UiT), Tromsø, Norway; 11 Directorate for Nature Management, Trondheim, Norway; University of Veterinary Medicine Hanover, Germany

## Abstract

The magnitude and urgency of the biodiversity crisis is widely recognized within scientific and political organizations. However, a lack of integrated measures for biodiversity has greatly constrained the national and international response to the biodiversity crisis. Thus, integrated biodiversity indexes will greatly facilitate information transfer from science toward other areas of human society. The Nature Index framework samples scientific information on biodiversity from a variety of sources, synthesizes this information, and then transmits it in a simplified form to environmental managers, policymakers, and the public. The Nature Index optimizes information use by incorporating expert judgment, monitoring-based estimates, and model-based estimates. The index relies on a network of scientific experts, each of whom is responsible for one or more biodiversity indicators. The resulting set of indicators is supposed to represent the best available knowledge on the state of biodiversity and ecosystems in any given area. The value of each indicator is scaled relative to a reference state, i.e., a predicted value assessed by each expert for a hypothetical undisturbed or sustainably managed ecosystem. Scaled indicator values can be aggregated or disaggregated over different axes representing spatiotemporal dimensions or thematic groups. A range of scaling models can be applied to allow for different ways of interpreting the reference states, e.g., optimal situations or minimum sustainable levels. Statistical testing for differences in space or time can be implemented using Monte-Carlo simulations. This study presents the Nature Index framework and details its implementation in Norway. The results suggest that the framework is a functional, efficient, and pragmatic approach for gathering and synthesizing scientific knowledge on the state of biodiversity in any marine or terrestrial ecosystem and has general applicability worldwide.

## Introduction

The magnitude and urgency of the biodiversity crisis is widely recognized within scientific and political organizations [1]. However, the absence of integrated biodiversity measurement and monitoring tools [2], [3] has constrained the ability of national and international organizations to respond to the biodiversity crisis. Two main reasons have been suggested for this [3]. First, biodiversity is a highly complex concept encompassing different organizational levels, from genes to ecosystems, and variable spatiotemporal scales. Second, there was no organized structure for mobilizing the expertise of the large scientific community to inform governments, until the approval of the Intergovernmental Science-Policy Platform on Biodiversity and Ecosystem Services in June 2010, the Convention on Biological Diversity, and other international agreements concerned with biodiversity. No structure existed to bring together the expertise of the scientific community and regularly provide validated and independent scientific information on biodiversity and ecosystem services to governments, policymakers, international conventions, non-governmental organizations, and the wider public [3]. The volume and diversity of published results, reports, and popular media communications make the scientific community a highly disorganized information source [4]. The purpose of integrated biodiversity indexes is to reduce the complexity of information and facilitate information transfer from science to other sectors of human society [5]–[8].

Previous attempts to provide integrated measures of biodiversity have included GLOBIO [9], the Dutch Natural Capital Index (NCI) [10], and the South African Biological Intactness Index (BII) [11]. The principle of these indexes is to combine a range of landscapes with a measure of biodiversity in order to illustrate general changes in ecosystems and their species content. However, published studies fail to integrate aquatic, marine, and terrestrial environments within the same framework. Most rely on assumptions about relationships between land use and biodiversity, which limits their general applicability. The aim of the Nature Index (NI) framework, which was developed and first applied in Norway, was to provide a general, transparent, internationally transferable, and integrated monitoring tool for biodiversity measurement [12].

The NI framework collates tractable, calibrated, and scientific information on biodiversity and the state of ecosystems from a network of experts within all fields of biomonitoring and ecological research; this network is referred to as the Ecological Research Network (ERN). The framework synthesizes scientific information from diverse sources and presents it in a transparent form in order to improve accessibility for environmental managers, policymakers, and the public. The NI framework allows for the comparison, application, and traceability of information from any ecosystem type by optimizing the use of existing information by incorporating expert judgment and monitoring-based and model-based estimates to provide a scientific overview that assists environmental managers and policymakers to set monitoring priorities and objectives. This also facilitates the identification and quantification of the extent to which knowledge on specific areas or ecosystems is lacking, which is essential for optimizing research priorities. The network of scientific experts chosen to represent the ERN are each responsible for one or more biodiversity indicators. The resulting indicator set is believed to represent the best available knowledge on the state of biodiversity and ecosystems in any given area [13], [14]. Indicators refer to natural quantities related to any aspect of biodiversity. To aggregate this knowledge, the value of each indicator is scaled relative to a reference state, i.e., an expected value assessed by each expert for a hypothetical undisturbed or sustainably managed ecosystem. Scaled indicator values can be aggregated or disaggregated over axes representing spatiotemporal dimensions or thematic groups.

In this study, we present the NI framework and detail its implementation in Norway. The results suggest that the framework is an efficient approach for collecting and aggregating information on biodiversity and has potential applicability as a functional, efficient, and pragmatic general approach for gathering and synthesizing scientific knowledge on the state of ecosystems and biodiversity.

## Methods

### The Nature Index Framework

#### Definitions

In the NI framework, a biodiversity indicator is defined as [15]:

“A natural variable related to any aspect of biodiversity, supposed to respond to environmental modification and representative for a delimited area. It is a variable for which a value in a reference state can be estimated. The set of indicators should cover as homogeneously as possible all aspects of biodiversity, and any addition of a new indicator should result in the addition of information.”

Thus, a biodiversity indicator might refer to the density, abundance or distribution of a population of a single species, a taxonomic, functional or genetic diversity metric, a demographic or behavioural parameter, or any other natural parameter fitting the definition. Several indicator-based assessments of biodiversity or an ecosystem state emphasize the requirement for using a large number of indicators to ensure broad coverage of many aspects of ecosystems and biodiversity, i.e., structural, functional, and taxonomic levels [16], as well as providing a way to monitor different environmental pressure or the provision of ecosystem services [13], [17]–[21]. Designing a perfect set of biodiversity indicators might take decades [22]. Therefore, we adopted a pragmatic approach to building a set of biodiversity indicators that aggregated most of the knowledge available from the ERN [14].

The use of reference states in the NI framework responds to both theoretical and pragmatic needs. References provide a context for the interpretation of each observed indicator value, allowing all observed indicator values to be comparable on the same scale [11], [23]. A reference state has been defined as follows [15]:

“The reference state, for each biodiversity indicator, is supposed to reflect an ecologically sustainable state for this indicator. The reference value, i.e., the numerical value of the indicator in the reference state, is a value that minimizes the probability of extinction of this indicator (or of the species or community to which it is related), maximizes at least one measurable aspect of biodiversity of the natural system to which it is related, and does not threaten any measurable aspect of biodiversity in this or any other natural system.”

Thus, a “measurable aspect of biodiversity” refers to a biodiversity metric at a specified scale [24]–[26]. In practice, the expected value of an indicator in a reference state is used to scale the observed (or estimated) value of each indicator, thereby ensuring that all scaled indicator values are directly comparable. Scaling is a means of measuring the difference between the observed variable and the reference state.

The observed and reference states of a given indicator can be estimated from data, either by model prediction or by expert judgment. As in other approaches to biodiversity assessment [11], expert-based judgments allow the assembly of the maximum volume of information. A reference state can be defined specifically for each indicator, according to the current state of knowledge for each indicator and ecosystem. Indicators do not need to share the same reference state, provided reference states fit the definition above.

Natural systems are composed of a mosaic of ecosystems, and it is crucial that they are distinguished explicitly. Within the NI framework, natural systems are termed “major ecosystems” and are categorized into a set of nine broad natural system types, i.e., mountain, forest, open lowland, freshwater, mires and wetland, coast pelagic, coast bottom, ocean pelagic, and ocean bottom (see Table S1 for definitions). Most ecosystems fall into these broad categories, but other categories, e.g., desert and ice cover, or subdivisions, e.g., different types of forests, can be added as local conditions demand.

The design of spatial and temporal units must fit with the resolution of the available information and with the objectives of knowledge synthesis and management, which may vary among countries and regions. Our case study section details how appropriate units were specified for the implementation of the NI in Norway.

#### Nature Index calculation

The observed values, or “states”, 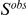 of indicator *i* belonging to major ecosystem *j* in spatial unit *k* at date *t* are denoted by 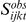. The corresponding values for the reference states are denoted by 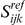. The same reference state for a given indicator can be applied to any date *t*. Both 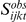 and 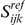 are non-negative values.

The estimate of the observed state for an indicator is assumed to be randomly drawn from a statistical distribution *L*, with two parameters *a* and *b*:
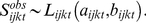
(1)Three forms of uncertainty can be considered in the NI framework: numerical uncertainty, data source uncertainty, and uncertainty because of lack of knowledge. Numerical uncertainty refers to uncertainty about the observed value of each indicator, which includes natural variability and observation uncertainty. Numerical uncertainty is taken into account when estimating 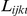. Monte-Carlo simulations can be implemented to obtain 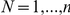 replications of the data collection process, which are denoted by 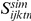. Estimating the set 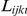 and implementing a simulation protocol to emulate authentically the data collection process is necessary to obtain a suitable measurement of numerical uncertainty. The case study section details how these problems were solved during the implementation of the NI for Norway.

Uncertainty because of the data source can be quantified by comparing the number of monitoring-based or model-based estimates with the number of expert-based estimates. This allows an assessment of deficiencies in the monitoring data set produced by the ERN.

In some cases, knowledge is so sparse that even expert-based judgments cannot be obtained. The number of documented indicators per spatial unit *k* provides a means of quantifying this lack of knowledge, which corresponds to the third level of uncertainty.

Each indicator can be expressed using a specific measurement unit, e.g., density, abundance, or species richness. Units must be scaled prior to averaging across spatial units or major ecosystems. Simulated indicator values 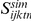 are scaled using their respective reference state value 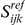. This gives a dimensionless quantity ranging from 0 to 1, where 0 is a completely degraded situation and 1 is an optimal situation for biodiversity, which corresponds to the chosen reference state.

**Figure 1:**
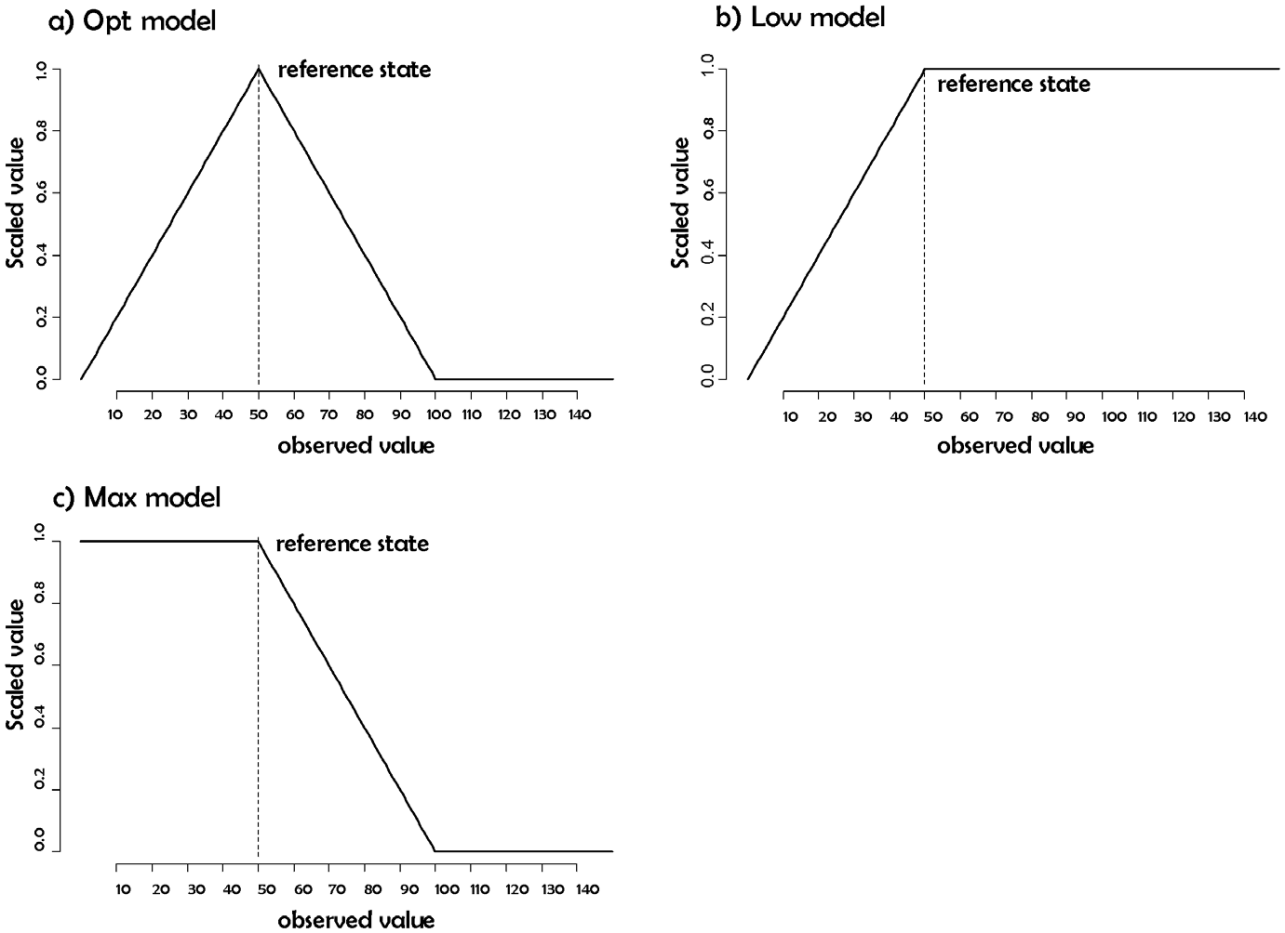
Examples of the use of scaling models. Scaled value when the observed value of a hypothetical indicator ranged between 0 and 150 and when the value in a reference state was 50.

Three simple scaling models were used to account for different ways of interpreting an observed indicator value relative to the expected value in a reference state (Figure 1).

The “optimal” model (Figure 1a) is defined as:
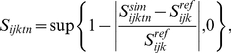
(2)where 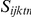 is the set of scaled simulated indicator values, i.e., a set of dimensionless values expressing the deviation of the observed indicator value from the reference state. The optimal scaling model implicitly assumes that any departure from the reference state results in a degradation of the state of the major ecosystem related to the indicator. This is useful for indicators such as the moose, *Alces alces*, which might experience a strong decline because of hunting but whose large populations have on the other side a detrimental effect on the vegetation because of an unsustainable grazing pressure [27], [28].

We use the “minimal” scaling model (Figure 1b) when the reference state refers to a low, precautionary level, as found in marine management of small pelagic fish [29]:
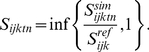
(3)When scaling the indicator for the minimal model, we assume that a deteriorated state for the indicator corresponds to a decrease below the reference level, and that any value above this reference level corresponds to an optimal situation.

We use the “maximal” scaling model (Figure 1c) when the reference state refers to a maximal value above which detrimental effects on ecosystems are observed, such as a maximal limit for the density of a proliferating species, or community, of phytoplankton or jelly-fish:
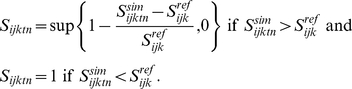
(4)Once the set of scaled indicators 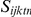 is calculated, it can be averaged across any of its axes *i*, *j*, *k*, or *t*, or any combination of axes. For example, an averaged value for all indicators, all spatial units, and all major ecosystems over time can be expressed as:
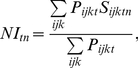
(5)where 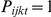 is a documented value for the indicator *i* in ecosystem *j* in spatial unit *k* and date *t*, and 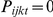 otherwise. 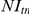 corresponds to a set of *n* simulated 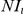 values, at date *t*. The final NI value can be expressed as the median of the simulated values, together with 95% confidence intervals around the median expressed as 2.5% and 97.5% quantiles. The set of simulated NI values allows for statistical testing by calculating *p*-values; for example, when comparing the index for two dates *t_1_* and *t_2_*, 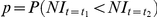.

#### Definition of weights

In previous implementations, no particular weights were applied to any of the *i*, *j*, or *k* axes. All calculations were made under a “complete equivalence” assumption, i.e., no locality, no major ecosystem, and no indicator was considered more important than another. This assumption is clearly open to criticism. If all components of biodiversity were equally studied, all indicators could be documented at all dates and spatial locations and, if all spatial locations were equally representative, there would be no need for weights. However, no matter how much care is taken when building the indicator set, discrepancies are likely to occur because not all taxa, functional groups, or geographical areas can be studied to the same degree [14], [20], [30]. Taxa such as fish, birds, and mammals are better documented than others, either because they attract more public interest or because study models are readily accessible. These potential discrepancies between spatial units or indicator representativeness meant it was necessary to introduce weights [14]. Weights can be defined across the indicator axis *i*, the major ecosystem axis *j*, and the spatial unit axis *k*. Introducing any set of weights *W_ijkt_* within the NI formula is straightforward:
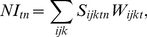
(6)where the condition 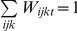 for any date *t*, and 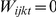 if indicator *i* has not been documented for the major ecosystem *j* in spatial unit *k* on date *t*.

The following rules for weights definition have been implemented in Norway. They have been designed to be readily transferrable to other countries with different data availability.

**Figure 2:**
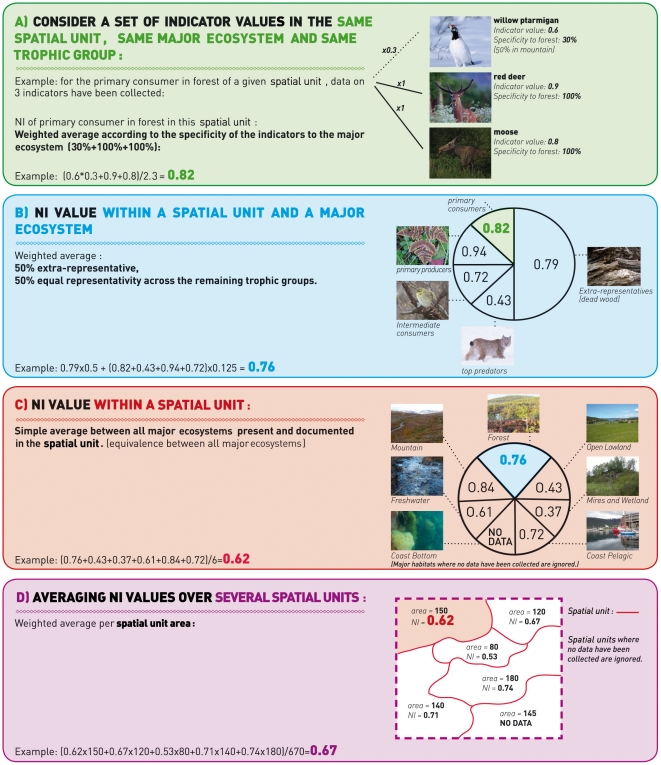
Simplified example of the Nature Index calculation process, including the weights used. For the sake of simplicity, the numbers of functional groups and major ecosystems have been slightly reduced relative to the Norwegian application.

Our approach addresses the following heterogeneities: indicators specific to a given major ecosystem versus indicators representative of several major ecosystems; indicators belonging to different taxonomic, trophic, or functional groups; well-documented indicators identified by the ERN as strongly representative of any aspect of biodiversity; and spatial units of different size. The following four sequential steps are used to control for these potential heterogeneities (Figure 2).

- At the finest level (Figure 2a), indicators for a group in a major ecosystem *j* with spatial unit *k* should be weighted according to their specific relationship to the major ecosystem using a relative measure of how this indicator relates to each ecosystem. For example, an indicator exclusively representative of forest, such as moose, *Alces alces*, receives a basic weight of 1 in a forest, but 0 in other major ecosystems. In contrast, the willow ptarmigan, *Lagopus lagopus*, is a representative of mountains and forests, where it receives a weight of 0.7 for mountains and 0.3 for forests.
- At the level of a major ecosystem *j* within a spatial unit *k* (Figure 2b), some indicators can be considered as particularly important indicators because their values strongly correlate with the state of the ecosystem. The contribution of these “extra-representative” indicators is set at a maximum of 50% of the NI value per spatial unit to ensure that they contribute significantly to the NI value but to prevent them from overwhelming information from other indicators. The following criteria were applied to the selection of extra-representative indicators: (i) they are representative of many species, (ii) they are representative of a large area encompassing several spatial units, and (iii) they are documented by data that allow estimation of the indicator for multiple dates and for the reference state. The other indicators should be weighted such that different groups contribute equally to the NI value, when the NI is calculated for each spatial unit of a major ecosystem (Figure 2b). In our example, the groups are trophic groups. The definition of groups may depend on the knowledge available from the ERN.
- At the spatial unit *k* level (Figure 2c), all major ecosystems *j* assumed to be present in a spatial unit are given equal weights. We assume that each major ecosystem holds a unique spectrum of biodiversity, which prevents them from being ranked against each other. Weights must be calculated to ensure equivalence. In contrast to the BII, this rule ensures that the NI is robust against change in land use [31]. If any major ecosystem is destroyed, the NI value will decrease until the same major ecosystem is restored.
- To aggregate across several spatial units (Figure 2d), weights should be allocated according to the area of the spatial unit *k* to ensure that any set of NI values averaged over several spatial units is representative of the total area. In our example (Figure 2), the spatial units were municipalities that differed in area.

The rules for calculating the weights are based on three criteria: (i) some indicators are known to be of higher importance to biodiversity, (ii) indicators can be classified into groups of equal importance in a major ecosystem, and (iii) no major ecosystem is more important than another.

#### Presentation of results

NI results can be presented at several aggregated levels and the choice of resolution depends on the underlying question addressed. Presenting the NI as a single value averaged over the axes *i*, *j*, and *k*, may not be the best way to illustrate and synthesize results. Apart from communication purposes, the usefulness of such a global measure is of limited use in environmental management, where sub-indexes may be more relevant. Maps for a specific major ecosystem on a given date, or trends for a given major ecosystem over a specific area, are much easier to interpret and of greater utility to environmental management. Global maps showing average NI values for several major ecosystems may be useful for communicating to the public.

The flexible design of the NI framework lends itself easily to the development of sub-indexes (thematic indexes) that focus on given trophic, taxonomic, or threatened species groups in a specific region or on biodiversity pressures associated with a particular environmental problem. Weights attached to thematic indexes can be binary, in order to reflect the selection of the indicators, major ecosystems, and localities that are relevant to a given theme.

### Case Study: The Nature Index for Norway

#### Spatiotemporal resolution of the Nature Index for Norway

Data were collected in Norway for four years (1950, 1990, 2000, and 2010) using 430 Norwegian municipalities as spatial units (see Text S1 for more details on the practical implementation). Four large regions were applied to open oceans outside coastal waters: Skagerrak, North Sea, Norwegian Sea, and Barents Sea. We chose the year 1950 as our starting point, because data prior to that date were considered unreliable and we wanted to measure the biodiversity impact of strong economic growth during the post-war period. Intervals of 10 years since 1990 were selected to make a trade-off between the expected sensitivity of the index, the amount and quality of older data, and the amount of work required.

#### The selection of indicators

The task of identifying biodiversity indicators involved a succession of meetings, which were organized according to major ecosystems; experts selected indicators based on the NI definition and any additional criteria specifically required for the Norwegian implementation of the NI [32]. Experts were required to report several items of information related to each biodiversity indicator (detailed in [15]), including broad ecological characteristics of the indicator, information on conservation or management interest, and other factors affecting weighting and sub-indexing. The whole indicator set is available as an Excel table (Table S2). Information concerning the specificity of indicators to major ecosystems can be found in Table S2, columns P to X. Following discussions with the ecological reference group, weights were considered for eight groups (Table S2, column AH): primary producer generalist, primary producer specialist, decomposer of organic matter, primary consumer and filter feeder, intermediate predator specialist, intermediate predator generalist, top predator specialist, and top predator generalist. The distinction between generalist and specialist was made by each expert.

#### Data collection

Data collection began in late June 2009 and was completed in September 2010, before publication of the first version of the NI. Data were assembled via a website connected to an SQL database, which was hosted by the Norwegian Institute for Nature Research (NINA). A demonstration version of this website can be found at http://naturindeks.nina.no (optimized for Microsoft Internet Explorer). The “Veiledning” section of the website opens the manual used to guide experts through the process of data preparation and data entry. Experts used the website to enter the observed value for each indicator, by municipality and by date. Experts also entered the value of the reference state for each indicator in each municipality.

Operational definitions (Table S3) were provided to help experts estimate reference states. All these definitions conformed to a general template. Experts could enter “monitoring based estimates”, “model-based estimates”, or “expert judgments” for their data [11], [33], [34]. A specific field kept track of data sources. Experts chose the scaling model for their indicators (Table S2, column AW).

Experts had to provide lower (25%) and upper (75%) quartiles for each observed indicator value as a measure of numerical uncertainty, as suggested by [33]. Experts could explicitly report a complete lack of knowledge instead of reporting a value for each estimate, i.e., a combination of indicator, spatial unit, and date. When no data were entered, we assumed that the indicator was absent and that nothing was reported.

Geographical information system analyses were used to calculate total municipality area and the area of each major ecosystem within each municipality. GIS calculations were based on the major ecosystem definitions in Table S1, Norwegian digital topographic maps (scale 1∶50,000), and vegetation maps [35]. These calculations were used to identify municipalities with and without mountainous areas and to standardize the presentation of the NI results to match those found with the NCI [10], [12].

#### Estimating numerical uncertainty

We used three values to estimate the statistical distribution for each set *L_ijkt_*: the mean observed value of the indicator, and the associated lower and upper quartiles. The process of estimating the statistical distribution using this limited amount of information was very simple. Several statistical distributions were tested, depending on whether the indicator was a continuous or a discrete variable. We calculated the following criterion *C* for a given two parameters statistical distribution *L(a,b)*:
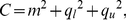
(7)where *m* refers to the difference between the observed mean estimate of the indicator and the mathematical expectation of the random variable following the distribution *L(a,b)*. The terms *q_l_* and *q_u_* refer to the differences between the estimated lower and upper quartiles of the indicator and the lower and upper quartiles of the distribution *L(a,b)*. For each observed indicator value 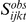, we retained the set *L(a,b)* that minimized *C*. We tested the following statistical distributions: for continuous variables, we tested truncated-normal, Gumbel, log-normal, Weibull, and gamma distributions; and for discrete variables, we tested Poisson, zero-inflated Poisson, and negative binomial distributions. Once the set *L_ijkt_* was identified, 999 simulated data sets were computed. These simulations mimicked the way data had been entered by the expert. In some cases, the same data were duplicated for several localities. The same simulated data vector was also duplicated for localities where data had been duplicated.

#### Presentation of Nature Index results for Norway

In the Norwegian case study, NI results were communicated as maps specific to each major ecosystem (steps a and b, Figure 2) and as trends averaged over the whole country (steps a, b, and then d, Figure 2), with confidence intervals. For mountain ecosystems, the NI calculation was restricted to municipalities where mountains comprised at least 20% of the municipality area. The remaining major terrestrial ecosystems were assumed to occur everywhere in Norway.

The mean number of indicators documented per municipality was calculated for each data source type (data, model, or expert), date, and major ecosystem to illustrate gaps in the data and to detect uncertainty because of data sources.

Some additional analyses were implemented and they are provided as supporting material. They concern the effect of our weighting system on the NI values (Text S2) and a convenient method for communicating NI results to the public, i.e., maps with averages across all indicators per municipality and major ecosystem (Text S3). The ability of the NI framework to focus on topics of environmental concern was demonstrated through four thematic indexes, i.e., top predators, freshwater acidification, environmental quality in the Oslo fjord, and trophic groups in pelagic ecosystems (Text S4).

#### Statistical and programming tools

The code used to calculate the NI is available as supporting information (File S1). Data processing and computations were performed using R 2.11.1 freeware [36]. The R code provided in File S1 shows functions used in statistical fitting, data simulation, NI computation, thematic index computation, mapping, and estimation of confidence intervals. The data set collected for mountains is provided as an example. The code in File S1 allows the user to make more specific plots than the ones we present, e.g., maps for each separate indicator (code S1, “NI commands.R” file, section 7.2), comparisons of interpolated and non-interpolated maps (section 7.5), or maps comparing changes over time with their associated *p*-values (section 7.7).

## Results

### The indicator set and associated reference states

A total of 308 indicators were selected by experts and used for calculations (Table S2). Of these, 238 were specific to a major ecosystem and 70 were representative of at least two major ecosystems. When these were duplicated into the major ecosystems they represented, the total indicator set was composed of 395 indicators. Table 1 shows clearly that the indicator set was extensive and covered many variables in the ecosystems; all variables were represented by at least one indicator in each major ecosystem. Documentation of these indicators at the municipality level for the sample dates of 1950, 1990, 2000, and 2010 and the reference state produced almost 300,000 database entries.

**Table 1:** Number of indicators per major ecosystem and thematic group.

	Tot	Spe	Key	Red	Comm	Serv	Ext
Ocean bottom	31	10	5	6	3	26	4
Ocean pelagic	40	16	7	7	2	32	5
Coast bottom	48	27	6	5	8	35	8
Coast pelagic	35	9	5	4	2	27	3
Open lowland	57	30	7	12	2	30	4
Mires and wetland	40	29	6	10	1	22	4
Freshwater	42	36	14	14	9	21	4
Forest	72	59	11	12	5	23	5
Mountain	30	22	7	6	2	16	3

**Tot**: total number of indicators. **Spe**: indicators specific to only one major ecosystem. **Key**: indicators related to a keystone species. **Red**: indicators related to vulnerable, endangered, or critically endangered species on the red list. **Comm**: indicators related to an ecological community. **Serv**: indicators related to the provision of ecosystem services. **Ext**: indicators considered as extra-representative by the experts.

Understanding how reference states were set across major ecosystems enhances our understanding of how inferences can be drawn from the indicator set (Table 2). For most terrestrial ecosystems, the majority of indicators refer to reference states established under “pristine or near-pristine natural conditions”. This was obvious in non-intensively harvested systems that were converted into more “productive” systems, e.g., mires and wetland, or when there was some access to almost pristine locations that served as a reference, e.g., forests, mountains, coast bottoms, and mires and wetland. “Pristine or near-preastine natural conditions” was viewed as a less important reference in several harvested ecosystems, including open lowland, coast pelagic, and ocean pelagic, where it was replaced by concepts of “traditional management” (open lowland), “precautionary level”, and “past knowledge” (marine ecosystems). The last two concepts were more frequent in marine ecosystems than in terrestrial ecosystems. This highlights the differences in research practice between these two areas, i.e., direct observations were more common in terrestrial systems, whereas most marine systems studies focused on long time series of indirect observations for stock assessment and management purposes. Resource management is a major issue in marine sciences [37], [38], which meant that many marine ecosystem reference states were related to precautionary harvesting levels, which were outputs of stock and recruitment-oriented demographic models. The use of prior theoretical or empirical indexes was restricted to freshwater systems, where the traditional research reference was the best possible value of these indicators [39], [40]. The concept of carrying capacity was used for a small number of indicators in most major ecosystems, except for mires and wetland, and mainly concerned well-studied indicators such as moose and salmon [41], [42].

**Table 2:** Number of indicators per major ecosystem and per operational definition used to define the reference state (see Table S3).

	CC	Sust	Past	Prec	Prist	Best	Trad
Ocean bottom	4	0	12	6	3	0	6
Ocean pelagic	2	0	17	15	3	0	3
Coast bottom	4	0	12	5	22	0	5
Coast pelagic	1	0	4	23	6	0	1
Open lowland	1	1	8	17	24	0	6
Mires and wetland	0	1	4	0	32	0	3
Freshwater	1	2	4	0	27	8	0
Forest	8	2	18	1	40	0	3
Mountain	5	0	5	0	20	0	0

**CC**: carrying capacity. **Sust**: maximum sustainable value. **Past**: knowledge of past conditions. **Prec**: precautionary level. **Prist**: pristine or near-pristine nature. **Best**: best theoretical values of indexes. **Trad**: traditional management (1850–1950).

### The state of biodiversity in Norway

**Figure 3:**
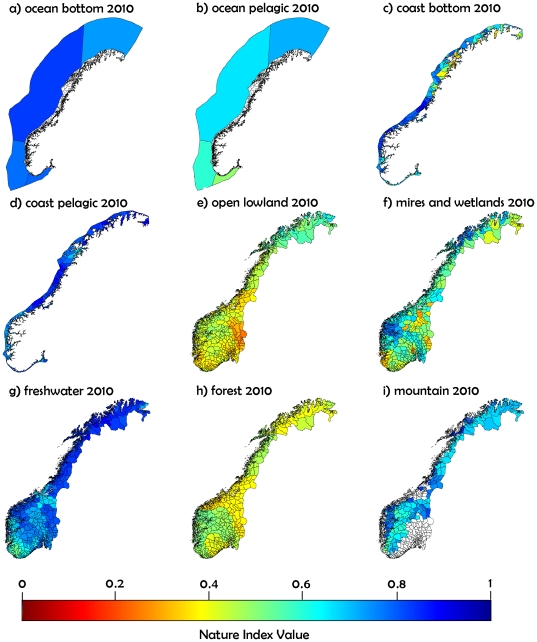
Nature Index values for each major Norwegian habitat in 2010.

**Figure 4:**
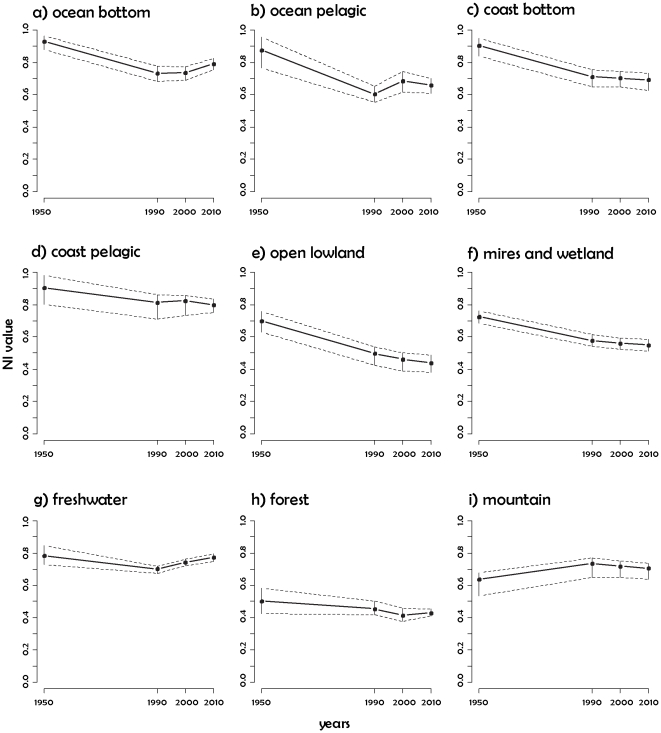
Trends in Nature Index values per major ecosystem, averaged over the whole of Norway. Grey lines and bars correspond to 95% confidence intervals.

The lowest Norway NI values for 2010 were found in open lowland, forest, and mires and wetlands (Figures 3 and 4), with NI values below 0.4 in some areas (Figure 3). NI values for ocean pelagic, coast bottom, coast pelagic, freshwater, and mountains ranged mainly between 0.5 and 0.8, depending on the area (Figure 3). Only the ocean bottom ecosystem was found to be in a good state, as assessed by experts. Trends for the major ecosystems (Figure 4) illustrate that most major ecosystems present had degraded NI values compared with their state in 1950. The confidence intervals were narrow enough to detect significant decreases (non-overlapping confidence intervals between two dates) in the case of ocean pelagic, ocean bottom, coast bottom, open lowland, and mires and wetland. In contrast, the freshwater NI values increased significantly from 1990 to 2010. The major ecosystems of forest, mountain, and coast pelagic presented non-significant trends. The lowest NI values for 2010 were reported for forest (mean = 0.43, confidence interval = 0.41–0.46) and open lowland (mean = 0.44, confidence interval = 0.38–0.49).

### Uncertainty because of data sources and lack of knowledge

**Figure 5:**
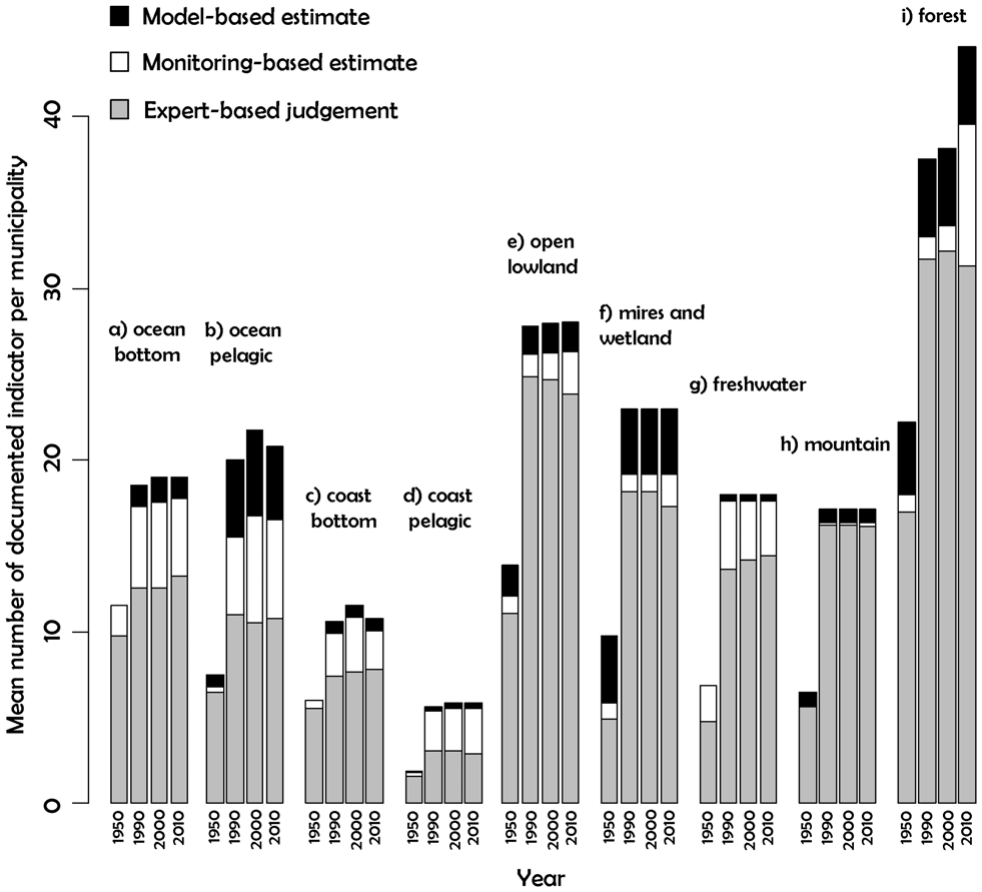
Mean number of documented indicators per municipality for each data source, date, and major ecosystem.

A high proportion of indicator values used for all systems were based on expert judgments (Figure 5). The proportion of expert-based estimates for marine systems was lower than for terrestrial systems. In contrast, the proportion of expert judgments was over 80% for major ecosystems such as mountains, open lowland, and freshwater. The high proportion of expert-based judgments for forests was balanced by a very high number of indicators documented per municipality and date. The number of documented indicators per municipality was lowest for coastal ecosystems. Fewer indicators were documented in 1950 compared with other dates for all major ecosystems. The mean number of indicators documented per municipality and date was compared with the total number of indicators for each major ecosystem (Table 1). For example, 35 indicators were defined for coast pelagic ecosystems, but only five were documented per municipality on average, which suggests that there is a huge margin for improvement in routine surveys in this major ecosystem.

## Discussion

### Interpreting the Nature Index

The concepts of biodiversity and ecosystem state are strongly linked and it is commonly accepted that ecosystems with high biodiversity in terms of species, functions, and structures, are more robust and resilient to environmental pressure, meaning they are more likely to provide ecosystem services to society [43]. Most indicators were closest to their reference state in areas with high NI values and we consider that these are areas where: (i) biodiversity is likely to be high relative to an ideal (reference) situation, and (ii) the ecosystem functioning is likely to be in a near optimal state, with high resilience and a satisfactory level of services provisioning, i.e., properties, goods, and services [44]. The NI results indicate the most likely state of biodiversity, given the knowledge that experts are able and willing to communicate.

By challenging experts to produce indicators with reference states estimated using the theoretical and operational definitions, we were able to synthesize a reference state for Norwegian nature. This ideal natural environment would contain no harvested stocks at risk of extinction. The abundance, density, biomass, or area of distribution of most of the species or communities would be close to pristine conditions or alternatively close to the carrying capacity of their respective ecosystems. Agricultural practices would sustain biodiversity and ensure the production of ecosystem services dependent on open areas. This multi-criterion definition reflects the complexity of both natural and societal systems that a framework such as the NI must consider [17]. A concept such as pristine nature cannot be applied uniformly to all major ecosystems because human society is a part of nature and the definition of pristine nature deliberately excludes the impact of human society on natural systems.

Discrepancies in reference states must be considered when interpreting NI values. For example, a large number of forest indicators used the concept of pristine nature as a reference, but this concept was rarely used in oceanic areas (Table 2). Direct comparison of these two major ecosystems using NI values must be conducted with caution, keeping in mind that their respective reference states are directed toward two different situations, i.e., sustainable harvesting (ocean) and an untouched natural system (forest). The design of new indicators must consider this issue. The addition of indicators related to pristine nature in the case of ocean and indicators related to sustainable harvesting for forest should be considered to control for these heterogeneities.

Not all reference states are directed toward exactly the same situation, but they provide environmental managers with a comprehensive set of reference levels when comparing potential goals and objectives. The optimal biodiversity definition needs not necessarily coincide with an optimal definition from an environmental management or political perspective. The distinction between reference states and management objectives is a crucial aspect of the implementation of the NI framework for management and policy purposes. For instance, management objectives might differ from the reference value in the case of trade off between biodiversity and other needs in the society.

The Norway NI shows that several ecosystems are under threat. In 2010, only three major ecosystems (ocean bottom, coast pelagic, and freshwater) were estimated to be in an overall good state with an NI around 0.8 and with the lower end of the confidence interval still above 0.7 (Figure 4). All other major ecosystems showed lower values, either in specific areas such as mires and wetlands, or over whole territories, such as forest or open lowland (Figure 3). In well-studied systems the confidence intervals were narrow, which allowed us to detect trends, such as the significant improvement in the state of freshwater since 1990, which was probably because of reduced acidification pressure and management programs. In other less well-studied and highly variable systems, the width of the confidence intervals was larger, but still narrow enough to report a significant decrease in the state of ocean bottom, ocean pelagic, coast bottom, open lowland, and mires and wetland compared with the situation in 1950. The values for forest were relatively stable from 1950, as expected in a highly managed ecosystem. The trend for open lowland was strongly negative, which suggests a rapid degradation in its state. The number of indicators available for forest was high, which suggests that improved management and conservation actions are more important than increased monitoring. In ecosystems such as ocean, coast, or mountains, the confidence intervals were wide and trends unclear, indicating that increased research and monitoring efforts in these ecosystems would be beneficial. Both research and management actions are critically needed for open lowlands.

Spatial patterns in NI values (Figure 3, Text S3 and S4) were also informative. A predominant characteristic was a north–south gradient in biodiversity state, with northernmost areas considered to be in a better state (ocean pelagic, open lowland, mires and wetland, freshwater, and mountains). This north–south trend may be related to processes such as acidification of freshwater, and mires and wetlands [45]–[47] (Text S4) and to a generally lower human pressure in the north. Early abandonment of traditional land use, and the introduction of intensified agricultural practices, particularly affected southern areas and led to a decrease in open lowland biodiversity [48], [49]. Southern ocean pelagic ecosystems also suffered more from overharvesting, especially in the North Sea and the Skagerrak. The spatial pattern for the coastal bottom, which was most degraded in areas in the North and the centre of the Norwegian coast, was mainly explained by a change in benthic communities related to overgrazing of kelp by sea urchins [50], [51]. The central part of Norway was the most degraded for forest because this is the area where logging activity is focused. The NI framework highlighted specific areas where management actions are critically needed, including open lowland and mires and wetland ecosystems (Figure 3). Results obtained for the thematic indexes (Text S4) demonstrate the flexibility of the NI approach using specific case studies.

Much more information has been extracted from the Norwegian NI framework case study than the figures presented in this paper. The complete set of results is available and thoroughly discussed in [12]. When possible, interpretation of the NI has been achieved jointly with independent monitoring of the data. This is a recommended practice, which leads to a refined interpretation of the results and a good acceptance of NI conclusions by both scientists and managers.

### Methodological concerns

The NI is clearly related to the Dutch Natural Capital Index [10] and the South African Biological Intactness Index (BII) [11], but with important conceptual differences. The NI allows the combination of several types of reference states and does not rely on an assumed relationship with an environmental covariate, nor is it constrained by the availability or properties of this covariate, i.e., errors, spatiotemporal extent, and scale [52]. The importance of a major ecosystem is not proportional to its area and all major ecosystems are considered equal in terms of their importance and contribution to overall biodiversity. This prevents changes in land use management from artificially increasing the NI value [31]. Any general implementation of the NI framework would provide the scientific community with relevant and easy-to-use data on which predictive models could be built [9]. For example, it allows the testing of the effects of population density, environmental pressure, or poverty levels on the NI value, thereby opening the way for forecasting and scenario testing, which is an expected use of similar approaches [9], [11].

Heterogeneities in the indicator set often mirror heterogeneities in knowledge present within the ERN. A weighting system that controls for these heterogeneities was required. The true states of ecosystems are unknown, and so assessing the relevance of our weighting system appears challenging but this will be an important task in the near future. Comparison between weighted and unweighted NI calculations (Text S2) demonstrates that the only substantial observed effect of our weighting system was a decrease in the NI value for some major ecosystems. This emphasis on degraded states is probably because of the importance given to indicators identified as extra-representative by experts. As these indicators reflect trends for many species, they often present low values when compared with indicators that are only relevant in isolation, which might explain the reduction in NI values with weighting. In addition to controlling for discrepancies in the indicator set, our weighting system allowed a precautionary approach by reducing the risk of missing a decrease in the state of a major ecosystem. The weighting system also enabled standardization in the use of the NI. The NI framework could be implemented in two different areas by two independent teams, and the two resulting indicator sets are likely to differ. However, using the same weighting rules ensures standardization of the aggregated results. This facilitates comparison of NI values among areas, e.g., countries, even if different sets of indicators are used. Finally, as the number of indicators increases in a given area to cover all ecosystem components more extensively, their respective weights will become more and more similar. This property may be used as a guideline when selecting new indicators.

The development of the NI framework was based on a strong, cooperative process between scientists, managers, and the NI core team. Definitions and explanations are provided to the experts, but they were entirely free to choose which information they enter in the database. The NI core team relied entirely on the information entered by the experts. Creating reciprocal relationships of trust and other confidence-building measures between the NI core team and the experts (Text S1) was crucial for the NI framework [53]. Discussions and deliberations at all stages of the process were essential. Exchanges between the NI core team, the ecological group, and the experts were intense during our practical implementation, especially during the validation stage, which resulted in a real increase in trust and confidence between the experts and the NI core team. This process ultimately led to a better acceptance of the results by all parties, scientists, managers, and the public.

The inclusion of expert-based judgments was useful because it allowed us to cover information that was previously neglected or only used implicitly. Taken individually, any expert-based approach is more likely to be biased compared with a more classical, empirical approach, provided that the latter is conducted properly. Using a high number of experts is one way to control for these biases. Calibration experiments with similar expert-estimate collection processes showed a reasonable accuracy for expert performances [11]; however, it is likely that expert-based judgments result in increased uncertainty [54]. In the long run, calibration should be used to assess the relevance of expert-based judgments, e.g., simultaneous collection of expert estimates and field data [33]. Calibration would also allow the measurement of the bias associated with each expert-based judgment, which may differ according to the expert and the indicator considered. Further analytical developments could also consider the use of a Bayesian framework, which is extremely efficient for combining expert, monitoring-based, and model-based estimates and for updating existing knowledge on uncertainty. Such an approach was considered but was not implemented for the sake of simplicity because our Monte-Carlo approach was easier to implement and communicate.

### Implementation and utility of the Nature Index

The NI framework can be viewed as an operational and pragmatic reply to calls from the scientific community for the establishment of a general framework to monitor biodiversity [5]–[8]. The simple methodological background and statistical formulation makes the NI easy to apply in any context. Almost any type of natural metrics can be included within the NI, but choices must be made by experts. The experts chose how to express their biodiversity indicators, defined the reference state for each indicator, and then chose how to express the observed state relative to the reference state (the scaling model). This sequential process allowed the incorporation of scientific expertise and took into account the specificity of each indicator when summarizing indicators in a scaled measure. This approach greatly facilitated the analysis and interpretation steps and it contrasts with databases where non-directly comparable data are stockpiled and are difficult to synthesize [55].

Using the national level as the operational scale of implementation of the NI makes sense. However, it is possible to build the NI at other scales if relevant indicators and experts can be identified. The framework is general enough so that several NI projects could be implemented simultaneously and then aggregated. Indeed, NI values make sense when compared with each other, and the aggregation of all information (steps a–d, Figure 2) to obtain a single value for an entire country would not be very informative. However, it might be useful if neighbouring countries provide a similar measure.

Reporting on the state of biodiversity can help to clarify questions relating to the causes of change or the consequences of management actions, and it supports the development of monitoring programs directed to investigating the causes of observed declines [56]–[58]. Stakeholders can use the NI to quantify objectives in terms of nature management and conservation, e.g., keeping the NI value of a given ecosystem above a certain threshold [59]. Improved information on uncertainty and research needs would be valuable. In some major ecosystems, routine surveys in the field cover a very limited number of indicators and sites [60] and the NI framework allows their easy identification (Figure 5). Research objectives can be defined to compensate for these heterogeneities. Useful guidelines for the design of future research and management programs in Norway might include increasing the number of documented indicators for each major ecosystem to a minimum of 20 per municipality (as currently found in four major ecosystems, Figure 5) and reducing the proportion of expert-based judgments to 50% of the total in all major ecosystems (typically greater than 80%, Figure 5).

### Conclusions

Reducing the complexity of information may lead to over-simplistic schemes [61]–[62], but it is the key to increased information transfer [4]. Our experiences of implementation in Norway suggest that the NI framework provides an efficient and operational trade-off between these two needs.

The NI satisfies the expectations of the international community [63] and presents the key properties required for establishing milestones in ecosystem management. The NI clearly links the assessment process to communication with policymakers, improves data accessibility and operability, uses consistent indicator sets and reference points to guide the interpretation of biodiversity and ecosystem status and trends, and it provides an integrated ecosystem assessment system that gives information on the state of ecosystems rather than on individual areas. The definition of reference states is a challenging task, but it can be viewed as a catalyst for the ERN by raising new and inspiring questions about the meaning of the observed state of the indicators relative to the state of the ecosystems. As soon as new scientific results are available, the reference states can be updated to improve constantly the relevance of the NI. In Norway, the NI will be updated every five years.

The use of thematic indexes provides information on well-defined topics of societal interest, and prevents the NI from being a general and abstract measure. The explicit measure of uncertainty and the identification of gaps in knowledge are key elements for informing management and directing funding to future research needs. The application of the NI framework to other countries would be straightforward.

Given the high international concern about biodiversity loss at the global scale, a framework such as the NI, if widely applied, has the potential to contribute significantly to the estimation of trends in biodiversity and to the design of corresponding management policies, thereby increasing the efficiency of the societal response to the global threat to biodiversity.

## Supporting Information

Table S1 Definitions for the 9 major ecosystems used within the NI framework.
(PDF)

Table S2 Excel file. Detailed list of indicators collected for the NI project in Norway.
(XLSX)

Table S3 Examples of practical definitions that can be used to estimate the value of indicators in a reference state.
(PDF)

Text S1 Practical implementations of the Nature Index.
(PDF)

Text S2 Effect of the weights on the Nature Index calculation
(PDF)

Text S3 Evolution through time of NI values per municipalities averaged across oceanic, coast and terrestrial major ecosystems.
(PDF)

Text S4 Development of thematic indexes within the NI framework
(PDF)

File S1 R source code for the implementation of the NI. As an example, data on the indicators related to mountains in Norway are provided. The file can be decompressed with Winrar.
(RAR)
