# Supplementary material for: The Nature Index: A General Framework for Synthesizing Knowledge on the State of Biodiversity
Source: PLoS One. 2011 Apr 22;6(4):e18930. doi: 10.1371/journal.pone.0018930 (PMC3081300; doi:10.1371/journal.pone.0018930)
Supplement: Table S1 — Definitions for the 9 major ecosystems used within the NI framework. (PDF) [file pone.0018930.s001.pdf]

Table S1. Definitions for the 9 major ecosystems used within the NI framework.

Major ecosystem:	Description
mountain:	Open area above the actual forest line, including Tundra, arctic, alpine and sub-alpine shrub, shrub or herbaceous vegetation, open space with little or no vegetation above the forest line
forest:	Any woodland or wooded land
open lowland:	Open spaces below the actual forest line including shrub heathlands and semi-natural grassland (meadows and pastures), lands dominated by forbs, mosses, lichens, Temperate shrub heathland, Inland unvegetated or sparsely vegetated habitats, coastal cliffs
mires and wetland:	Mires, bogs, fens, springs, flooded freshwater plains
freshwater:	Rivers and lakes, inland surface waters
coast pelagic:	Intertidal areas, Coastal marine area (<1 nautical mile off the coast), estuarine areas, excluding benthic fauna and flora
coast bottom:	benthic fauna and flora of the marine area located <1 nautical mile off the coastline
ocean pelagic:	Pelagic area in the Exclusive Economic Zone, that is outside the coastal zone and within 200 nautical miles
ocean bottom:	Benthic area in the Exclusive Economic Zone, and abyssal areas that are outside the coastal zone and within 200 nautical miles
