# Supplementary material for: The Nature Index: A General Framework for Synthesizing Knowledge on the State of Biodiversity
Source: PLoS One. 2011 Apr 22;6(4):e18930. doi: 10.1371/journal.pone.0018930 (PMC3081300; doi:10.1371/journal.pone.0018930)
Supplement: Table S3 — Examples of practical definitions that can be used to estimate the value of indicators in a reference state. (PDF) [file pone.0018930.s003.pdf]

Table S3. Examples of practical definitions that can be used to estimate the value of indicators in a reference state.

Name	Description
Carrying capacity	A theoretical value for a population number or density for example, according to the natural limit of a population set by resources in a particular environment.
Precautionary level	Recommendations provided by scientific and independent group of reflexion. Refers to a value below which the indicator, and therefore the major habitat to which it is related, is endangered
Pristine or near-pristine nature	An estimated value that refers to pristine, untouched or low impacted natural system
Knowledge on past situation	An estimated value derived from a known past situation, when the indicator was in good condition, and a situation that is always ecologically relevant today
Traditionally-managed habitat	A value observed under traditionally managed habitat, such as extensive, biological agriculture (e.g. pasture in open lowland). 1900 was chosen as a reference year for Norway.
Maximum sustainable value	A value below which no detrimental effects are observed for the major habitat to which the indicator is related.
Best theoretical value of indexes	If the indicator refers to an already developed index, such as a biodiversity index, its best (the value corresponding to the "best" state in term of biodiversity) expected value depending on the location and the major habitat
Amplitude of fluctuations observed in the past (for cycling of fluctuating species)	For fluctuating populations (typically rodents or small pelagic fishes): the amplitude of fluctuations over a given temporal windows that is observed in natural or low impacted conditions (specific case for pristine or past knowledge)
