# Supplementary material for: The Nature Index: A General Framework for Synthesizing Knowledge on the State of Biodiversity
Source: PLoS One. 2011 Apr 22;6(4):e18930. doi: 10.1371/journal.pone.0018930 (PMC3081300; doi:10.1371/journal.pone.0018930)
Supplement: Text S1 — Practical implementations of the Nature Index. (PDF) [file pone.0018930.s004.pdf]

Text S1. Practical implementation of the Nature Index

The process of implementing the Nature Index in Norway is illustrated in Figure S1a. The spatial resolution for data collection is illustrated in Figure S1b. The work has been coordinated by the "Nature Index core team", with support from an ecological reference group and a statistical reference group. The general framework has been developed in close collaboration with the scientists of the two groups. The ecological reference group and the Nature Index core team identified a list of "experts", mostly scientists involved in monitoring programs on relevant species or ecosystems in Norway. In total, 125 experts were involved, including the members of the ecological reference group. All belong to leading ecological research institutions, responsible for long-term monitoring of biodiversity in Norway. The network of experts is referred to as the Ecological Research Network (ERN). Each expert was familiarised with the NI general framework, and was asked to define one or several indicators according to our definition. Then, each expert entered observed and reference values of their indicators through an internet database. The spatial scale of municipality allows a fairly accurate location of data and is convenient for management. Alternatively, data could be entered at the county level (Fig S1b) and duplicated in all municipalities of a given county (e.g. for long-ranging birds). The choice of the spatial scale for data entry was left to each expert. The process of weight definition resulted from a consensus between the Nature Index core team and the ecological reference group. The ecological reference group suggested thematic indexes of particular national or management interest. During the validation stage, preliminary results were carefully checked by the ecological group and the experts, leading to numerous updates in the database. The final results in the form of maps or curves with confidence intervals were published and released to the stakeholders and the public [12].

Figure S1a. Description of the practical implementation of the Nature Index in Norway:

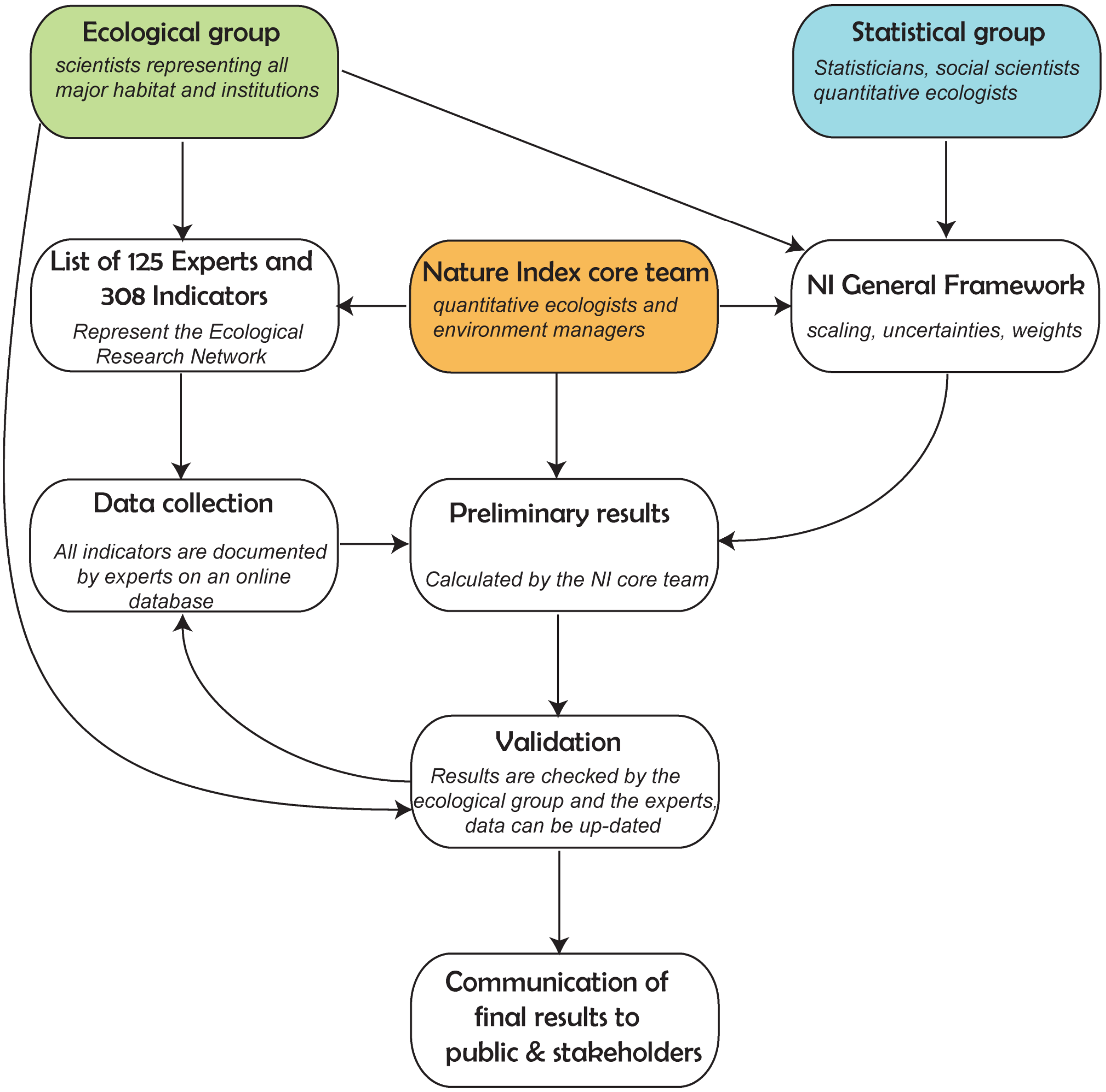

Figure S1b. Spatial scale of data collection: municipality, county and oceanic area

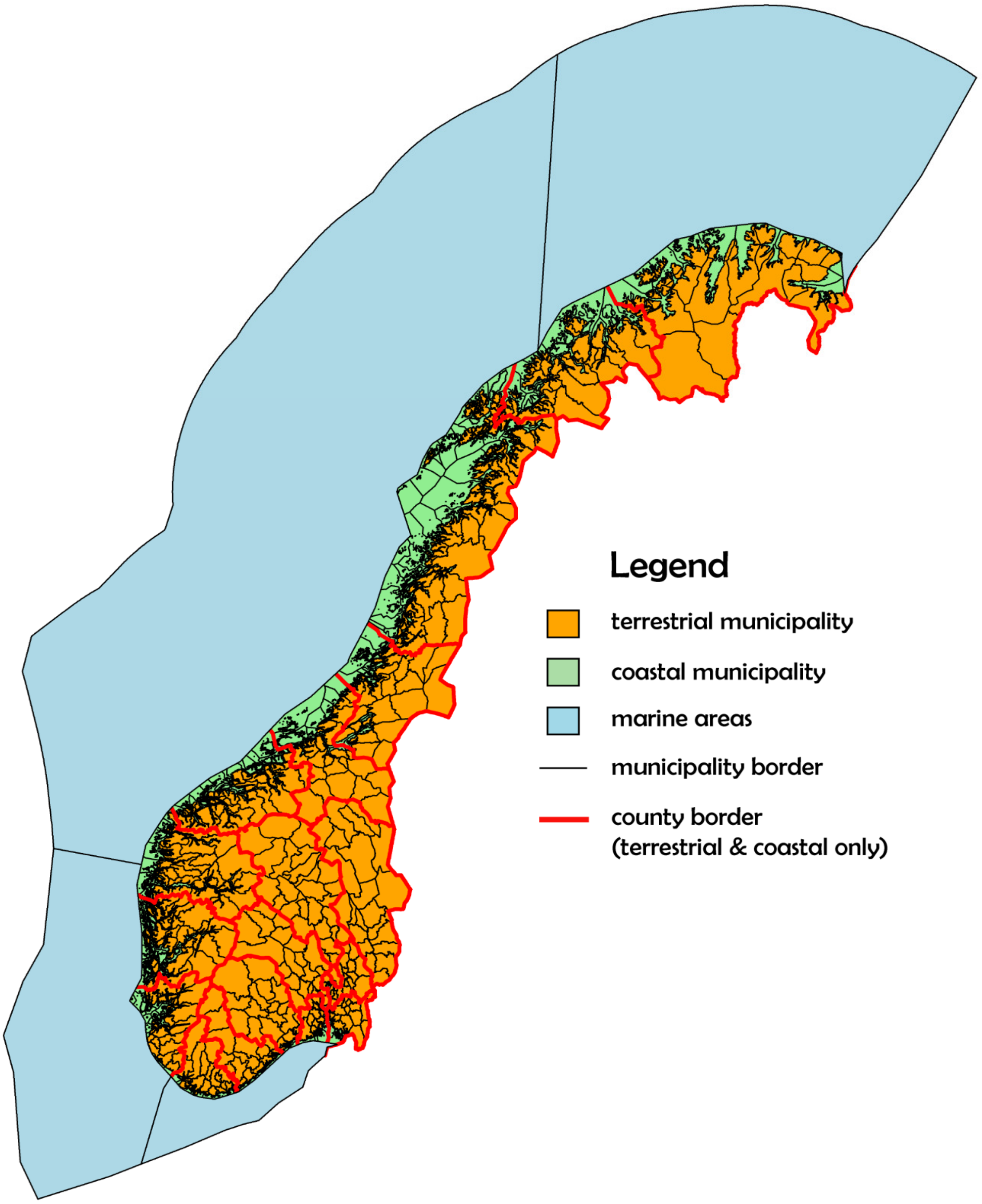

Basically, a team composed of a dozen of members can lead such a project. In the case of Norway, in full-time equivalent jobs for the years 2009-2010, two quantitative ecologists were responsible for methodological developments, data management and analysis and explanation of the framework to the experts. One scientist in the capacity of environmental

manager was responsible for networking activity, i.e. bringing expert groups together, and ensured the connection between scientists and managers. These three people constituted the NI core team (Fig S1a). The Ecological group was composed of nine scientists (one per major ecosystem), ensuring the link between experts and the NI core team and were responsible for data gathering and entering and for result interpretation. The Statistical group was composed of 6 statisticians, but its role was essentially consultative. The cost of the project will largely depend on the spatio-temporal resolution of the information on indicators. Collecting detailed information in space and time is time consuming and therefore costly, so the spatio-temporal resolution of the project can be tuned, if necessary, to cope with budgetary constraints. As the methodology and computer program are now available, as supporting material S8, we assume that when implementing the NI elsewhere, most work will be related to gathering data.
