# Supplementary material for: The Nature Index: A General Framework for Synthesizing Knowledge on the State of Biodiversity
Source: PLoS One. 2011 Apr 22;6(4):e18930. doi: 10.1371/journal.pone.0018930 (PMC3081300; doi:10.1371/journal.pone.0018930)
Supplement: Text S2 — Effect of the weights on the Nature Index calculation (PDF) [file pone.0018930.s005.pdf]

Text S2. Effect of the weights on the Nature Index calculation:

Fig S2: weighted (x-axis) vs. unweighted (y-axis) NI values per municipality.

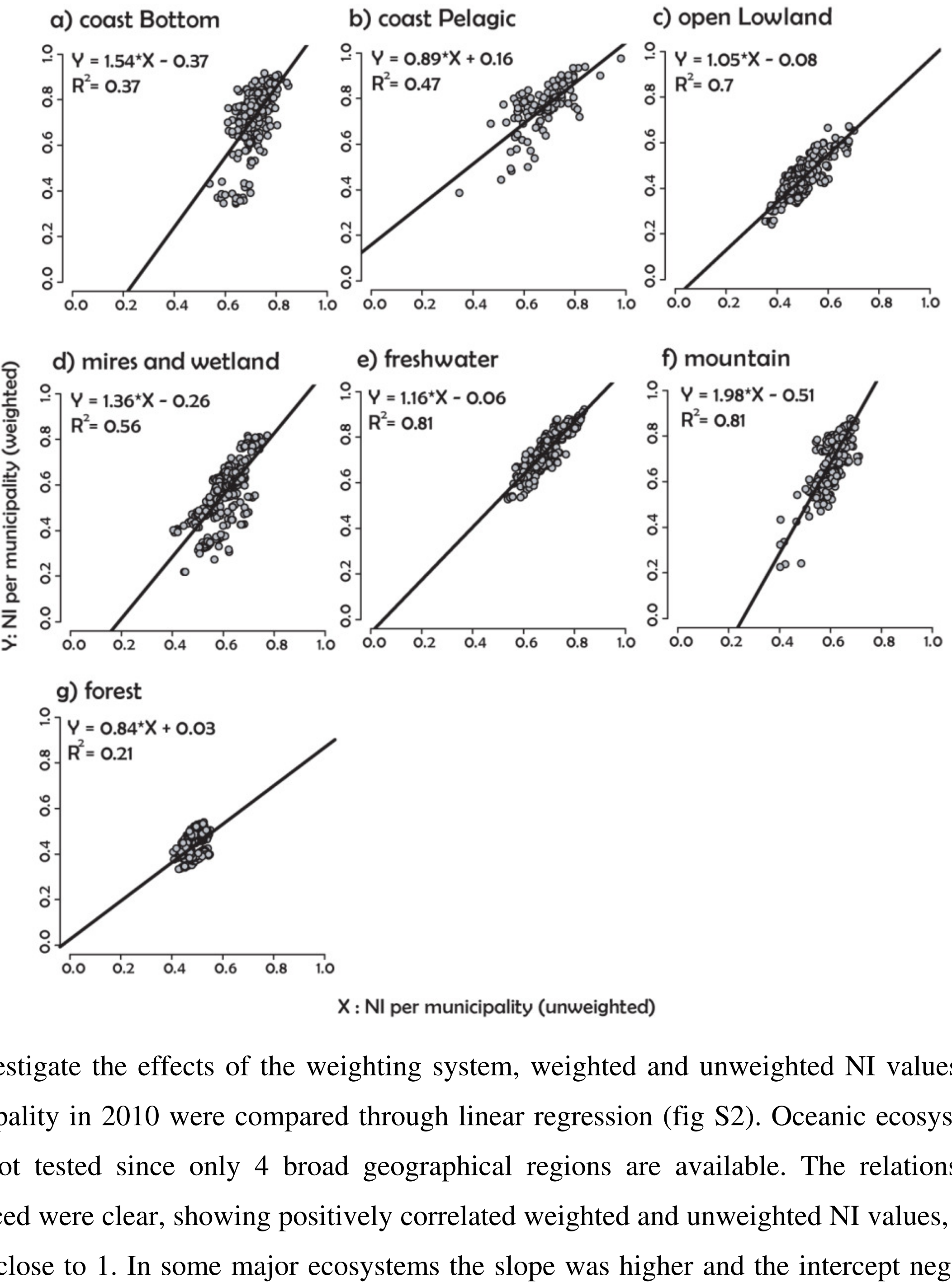

To investigate the effects of the weighting system, weighted and unweighted NI values per municipality in 2010 were compared through linear regression (fig S2). Oceanic ecosystems were not tested since only 4 broad geographical regions are available. The relationships evidenced were clear, showing positively correlated weighted and unweighted NI values, with slopes close to 1. In some major ecosystems the slope was higher and the intercept negative (coast bottom, mountain, and mires and wetland to a lesser extent). In these cases, the weights affected the low NI values that tended to be even lower. In coast pelagic the opposite was true: a slope of less than one and a positive intercept suggest that the weights increased the low NI values.
