# Supplementary material for: The Nature Index: A General Framework for Synthesizing Knowledge on the State of Biodiversity
Source: PLoS One. 2011 Apr 22;6(4):e18930. doi: 10.1371/journal.pone.0018930 (PMC3081300; doi:10.1371/journal.pone.0018930)
Supplement: Text S3 — Evolution through time of NI values per municipalities averaged across oceanic, coast and terrestrial major ecosystems. (PDF) [file pone.0018930.s006.pdf]

Text S3. Evolution through time of NI values per municipalities averaged across oceanic, coast and terrestrial major ecosystems.

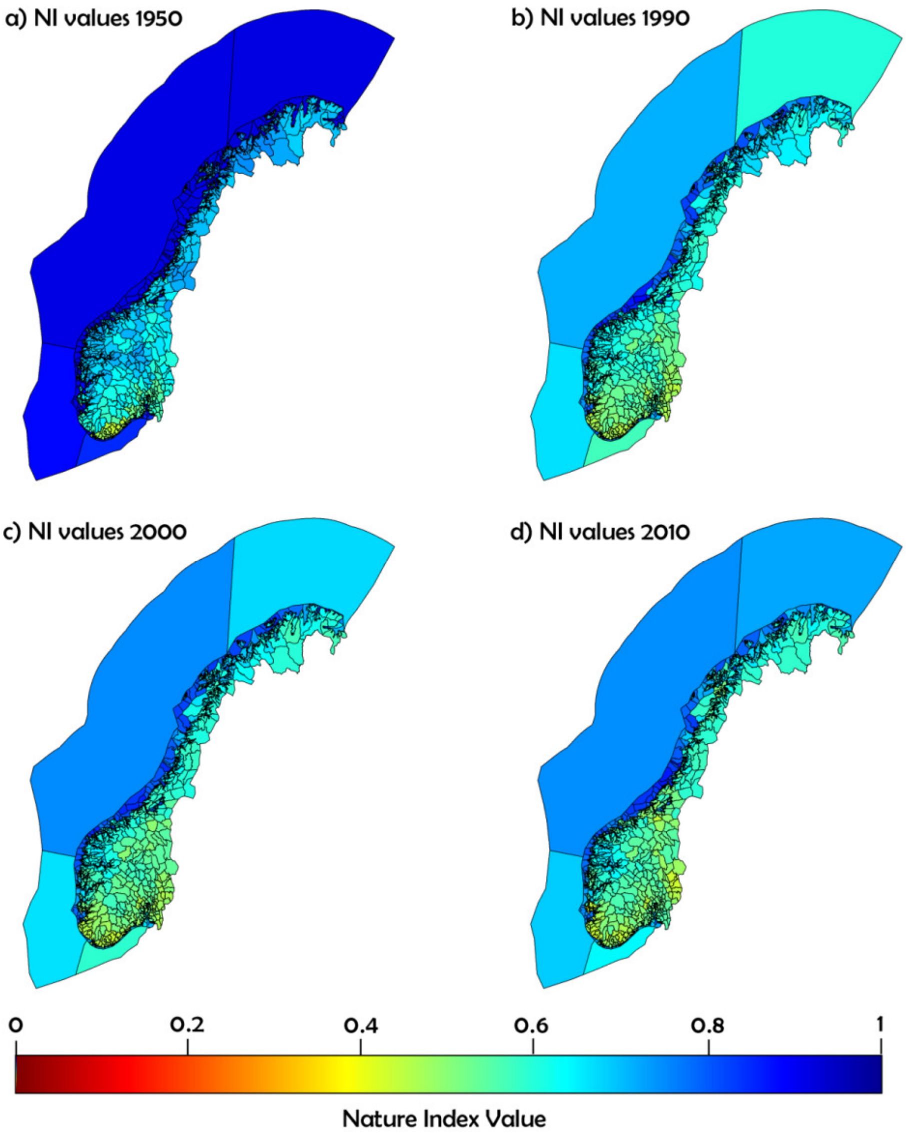

Integrating results for the public

To demonstrate the ability of the NI to make information on the state of ecosystems easy to present and interpret for the public, NI values over several major ecosystems were aggregated. The resulting overall maps are well suited to illustrate the decrease in biodiversity and in the state of ecosystems that has occurred in Norway during the second part of the 20th century, both in terrestrial areas (especially around the most populated places), but also in oceanic areas. Averaged maps may be less useful for managers who need to know which ecosystems are threatened.
