# Supplementary material for: The Nature Index: A General Framework for Synthesizing Knowledge on the State of Biodiversity
Source: PLoS One. 2011 Apr 22;6(4):e18930. doi: 10.1371/journal.pone.0018930 (PMC3081300; doi:10.1371/journal.pone.0018930)
Supplement: Text S4 — Development of thematic indexes within the NI framework (PDF) [file pone.0018930.s007.pdf]

Text S4. Thematic indexes

This section illustrates how the NI framework can be applied to well defined questions of management interest, through the use of four thematic indexes: on top predators, acidification in freshwater, environmental quality in the Oslo fjord, and trophic groups in pelagic ecosystems. When selecting indicators specific for a given theme, weights are binary, either 1 if the indicator is relevant for the chosen theme, or 0 otherwise. Then, all relevant indicators are simply averaged together over a spatial location. Weights for differences in the area of spatial units are still applied for thematic indexes.

For the first thematic index, all terrestrial and freshwater indicators belonging to the "top predator" group and with a specificity value higher than 50% to any major ecosystem were selected (Table S2, column AX). Steps a and c of Figure 2 were applied, and the resulting NI values in each municipality were calculated. The resulting maps (Fig S4a) indicate a poor state of top predator populations in Norway, especially in the southern part, but nonetheless suggest some improvement compared to the 1950 situation, especially in the northernmost areas.

The second thematic index focused on acidification in freshwater. A subset of indicators sensitive to this pressure was selected (Table S2, column AY) and simply averaged together for each municipality and date. The resulting maps (Fig S4b) showed a strong north-south gradient, with the southernmost part of the country being the most affected. However, NI values in the south in 2000 and 2010 suggested an improvement compared to 1950 and 1990.

The third thematic index focused on the Oslo fjord area. Previously calculated NI values for the coastal pelagic ecosystem are re-mapped at a smaller spatial scale to demonstrate the ability of the NI to provide local information. The zoom-in on the Oslo fjord of the NI values for the coastal pelagic ecosystem (Fig S4c) revealed a degraded situation in 1990 and 2000, with some improvement in 2010.

The fourth thematic index focused on oceanic areas. The subset of the indicators chosen corresponds to broad trophic groups in pelagic ecosystems (zooplankton, fish plankton feeders, fish predators, birds and mammals, Table S2, column AZ). We restricted the calculation of trends and confidence intervals to the three most recent dates (1990, 2000, and 2010) because very few data were entered in some groups for 1950. The resulting trends (Fig S4d) illustrated a recent decrease in the state of zooplankton, seabirds and mammals; a stable but low state for fish predators, whereas the state of planktivorous fishes increased, although

confidence intervals remain large. Differences in the state of planktivorous and piscivorous fish populations were significant in 2010.

Fig. S4: Four thematic index based on the NI framework for Norway

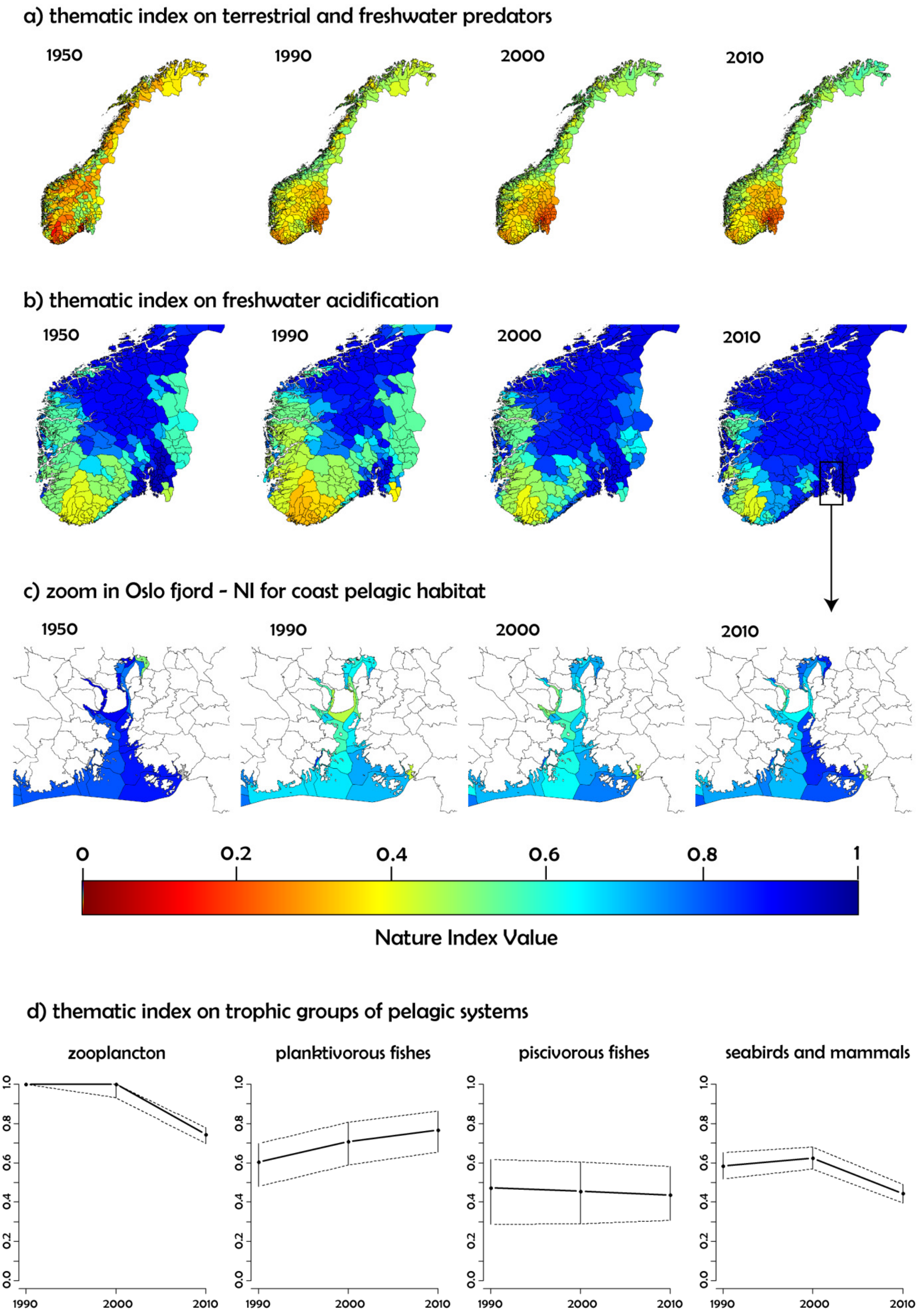

The relative improvement in the state of terrestrial predator populations from 1950 to today (Fig S4a) is in accordance with changes in management policies of the last decades [1,2] that led to conservation programs for mammalian carnivores (bear, wolf, lynx and wolverine, more recently also arctic fox). For birds, decrease in the use of pesticides, together with improved conservation measures, is at the origin of the recovery of several raptor populations [3]. However, absolute NI values for predators in 2010 are still low (0.2 – 0.5 depending on the area) suggesting that the current state of top predator populations is far from the reference state.

The thematic index on acidification (Fig S4b) illustrates the potential of the NI framework to document changes according to a given environmental pressure. In the second half of the 20th century, acid atmospheric deposition resulted in a great loss of ecosystem integrity and biodiversity in Norwegian freshwater systems, especially in the south [4]. However, a global reduction in sulphur deposition during the last decades relaxed the acidification pressure on freshwater ecosystems [5].

Zooming into the Oslo fjord area (Fig S4c) also demonstrates the potential of the NI framework to change of spatial scale. The Oslo fjord has a well documented history of cultural eutrophication from sewage discharge, culminating in the 1950s with massive hypoxia, fouling of recreationally important water resources, and collapse of a local fishery [6]. This was followed by a gradual reduction in nutrient loading as sewage treatment improved, starting in the 1960s through to 1982, when a new treatment plant further out in the system relieved the inner fjord of almost all such influence [7]. If this pattern is correctly represented in NI maps of the Oslo fjord for 1990, 2000 and 2010, the maps of 1950 failed to report the deterioration due to eutrophication. Indeed, there is very little information about the eutrophication status around 1950 that could be expressed by the selected indicators, e.g. no data for chlorophyll a. In addition, the number of documented indicators per municipality was extremely low for the coastal pelagic ecosystem (Fig 5d). This clearly highlights that the interpretation of NI values may lead to false conclusions when the number of indicators is low (<5). Such values have to be considered with caution.

Lastly, the trends of the thematic indexes of pelagic trophic groups (Fig S4d) fit well with the dominant patterns in harvested marine ecosystems where predator populations (predatory fishes, seabirds and marine mammals) are decreasing and the marine food web tends to be dominated by smaller, plankton-feeding fishes [8,9], resulting sometimes in increased grazing pressure on zooplankton.

1. Skonhoft A (2006) The costs and benefits of animal predation: An analysis of Scandinavian wolf re-colonization. Ecol Econ 58: 830-841.

2. Linnel JDC, Broseth H, Odden J Nilsen EB (2010) Sustainably Harvesting a Large Carnivore? Development of Eurasian Lynx Populations in Norway During 160 Years of Shifting Policy. Environ Model Softw 45: 1142-1154.

3. Gjershaug JO, Kålås JA, Nygård T, Herzke D, Follestad AO (2008). Monitoring of raptors and their contamination levels in Norway. Ambio 37(6): 420-424.

4. Schneider S, Lindstrøm EA (2009) Bioindication in Norwegian rivers using non-diatomaceous benthic algae: The acidification index periphyton (AIP). Ecol Indic 9: 1206-1211.

5. Fölster J, Wilander A (2002) Recovery from acidification in Swedish forest streams. Environ Poll 117: 379-389.

6. Dale B, Thorsen TA, Fjellså A (1999) Dinoflagellate cysts as indicators of cultural eutrophication in the Oslofjord, Norway. Est Coast Shelf Sci 48: 371-382.

7. Dale B (2009) Eutrophication signals in the sedimentary record of dinoflagellate cysts in coastal waters. J Sea Res 61: 103-113.

8. Planque B, Fromentin JM, Cury P, Drinkwater KF, Jennings S, et al. (2010) How does fishing alter marine populations and ecosystems sensitivity to climate? J Mar Syst 79: 403-417.

9. Perry I, Cury P, Brander K, Jennings S, Möllman C, et al. (2010) Sensitivity of marine systems to climate and fishing: Concepts, issues and management responses. J Mar Syst 79: 427-435.
